# Digestive Ferment in the Fig

**Published:** 1881-11

**Authors:** 


### DIGESTIVE FERMENT IN THE FIG.

M. Bouchut, who has been investigating the digestive principle of the papaw tree, has extended his researches to the common fig, and the result of preliminary experiments (*Comptes Rendus*, xci., p. 67), carried out upon the milky juice collected from a fig tree in April last, seems to show that this juice contains a powerful ferment capable of digesting albuminoid matters. As much as 90 grammes of fibrin, added in eight successive portions, at intervals of one or two days, to 5 grammes of the milky juice, and kept at a temperature of 50° C., was for the greater part digested, leaving a small amount of a white homogeneous residue, and the solution having the odor of good broth.

